# Survival analysis and factors affecting survival in patients who presented to the medical oncology unit with non-small cell lung cancer

**DOI:** 10.3906/sag-1912-205

**Published:** 2020-12-17

**Authors:** Özgür ÖNAL, Murat KOÇER, Hande Nur EROĞLU, Servet Derya YILMAZ, İsmet EROĞLU, Dilek KARADOĞAN

**Affiliations:** 1 Department of Public Health, Faculty of Medicine, Süleyman Demirel University, Isparta Turkey; 2 Department of Medical Oncology, Antalya Training and Research Hospital, Antalya Turkey; 3 Department of Internal Medical Sciences, Faculty of Medicine, Süleyman Demirel University, Isparta Turkey; 4 Department of Chest Diseases, Faculty of Medicine, Recep Tayyip Erdoğan University, Rize Turkey

**Keywords:** Non-small cell lung cancer, survival, factors affecting survival

## Abstract

**Background/aim:**

This study aimed to investigate the effect of clinical and pathological indicators at the time of the diagnosis on overall survival in patients recently diagnosed with non-small cell lung cancer.

**Materials and methods:**

The study population consisted of patients who were diagnosed at the Faculty of Medicine at Isparta Süleyman Demirel University Hospital between January 1, 2010 and December 31, 2017 and presented to the medical oncology outpatient clinic.

**Results:**

A total of 518 patients were evaluated, including 260 patients with squamous cell carcinoma, 207 patients with adenocarcinoma, 50 patients with non-small cell lung cancer-not otherwise specified, and 1 patient with large cell carcinoma. The average life expectancy was found to be 11.50 ± 1.40 months in patients with squamous cell carcinoma, 12.60 ± 1.59 months in patients with adenocarcinoma, and 8.70 ± 1.87 months in the other patients. The estimated 5-year relative survival rate for non-small cell lung cancer was 8% (7% for men and 18% for women). In the multivariate analysis, sex being male (HR, 2.41; P < 0.001), a performance status >2 (HR, 1.70; P < 0.001), the stage of cancer (HR, 1.37; P = 0.045), the presence of bone or liver metastasis (HR, 1.44, P = 0.009, HR, 1.57; P = 0.016, respectively), and the patient not having received radiotherapy (HR, 3.25; P < 0.001) or chemotherapy (HR, 1.85; P = 0.001) were defined as statistically significant predictive factors that reduced the overall survival.

**Conclusions:**

In this study, sex, stage, performance status, the presence of liver or bone metastases, and treatment had an effect on overall survival.

## 1. Introduction

Global evaluations show that the majority of deaths are caused by noncommunicable diseases. In predictions for the 21st century, cancer is expected to be the biggest obstacle to decreasing mortality rates and increasing life expectancy. According to the latest report published by the IARC (International Agency for Research on Cancer) based on the GLOBOCAN (Global Cancer Observatory) 2018 predictions, it was estimated that the global cancer burden would rise to 18.1 million new cases and 9.6 million deaths in 2018. According to this report, with 2.1 million new cases of lung cancer (11.6% of total cases) and 1.8 million deaths (18.4% of total cancer deaths), lung cancer continues to be the leading cause of cancer-related mortality and the most commonly diagnosed cancer in both men and women [1].

According to the cancer statistics report published by the Turkish Ministry of Health in 2014, tracheal, bronchial, and lung cancer is the most common type of cancer in men (21.1% (age-standardized mortality rate 52.5/100000)) and breast cancer is the most common type of cancer in women (24.9% (age-standardized mortality rate 43.0/100000)) in Turkey. According to this report, lung cancer is usually diagnosed at late stages and distant metastasis is observed in 52.7% of the patients at the time of the diagnosis [2].

Although improvements in early diagnosis and treatment with the hope of improving survival are promising, studies in the last 30 years have shown that the expected improvement in survival has not yet been achieved [3]. The 5-year survival rate for lung cancer is 18% (15% for men and 21% for women). Only 16% of the patients are diagnosed when the cancer is at a localized stage for which 5-year survival rate is 56% [4]. It has been the aim of many studies to determine the prognostic factors in order to better evaluate treatment efficacy and determine treatment methods in order to improve survival rates [5,6]. Ignoring these factors will prevent actual therapeutic differences from being revealed.

This study aimed to investigate the effect of clinical and pathological indicators on overall survival in recently diagnosed NSCLC (non-small cell lung cancer) patients and we believe that the standardization of these factors in future studies will help determine the optimal treatment.

## 2. Materials and methods

### 2.1. Study design

This study was conducted as a retrospective cohort study between 2018 and 2019. Approval was obtained for this study (no. 214, dated December 13, 2018) from the Ethics Committee of the Faculty of Medicine in Süleyman Demirel University.

**Figure 1:**
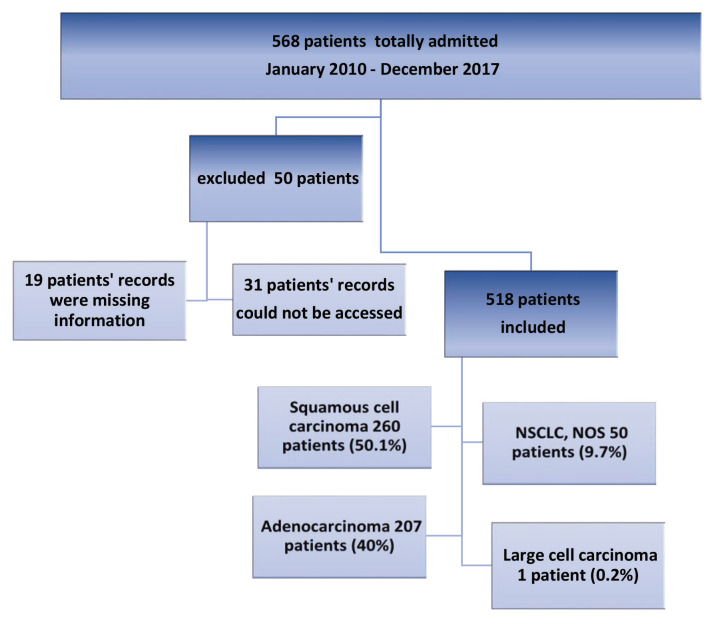
Number of patients admitted.

The study population consisted of non-small cell lung cancer patients who were diagnosed at the Faculty of Medicine at Isparta Süleyman Demirel University Hospital and presented to the Medical Oncology Outpatient Clinic between January 1, 2010 and December 31, 2017. Patient file records were reviewed by the researchers. It was aimed to study the entire population. Among a total of 568 lung cancer patients who presented to the oncology unit and received a pathological diagnosis, 518 patients (91.1%) whose records could be accessed were included in the study (Figure 1).

### 2.2. Data collection

Demographic data (age, sex, marital status, social security, comorbidity, history of alcohol use, and smoking), symptoms (presence of lung-related or non-pulmonary symptoms), clinical (performance status), and follow-up (treatments administered after diagnosis, follow-up duration, and overall survival rate, if any) information of the patients, and information related to the disease including the location of the tumor, histopathological subtype, stage, and metastasis site, if any, were recorded in the study.

### 2.3. Management of confounding variables

Age was evaluated as a continuous variable and according to a cut-off point of 60 years. Considering the symptoms of the patients at the time of the diagnosis, cough, sputum, dyspnea, and bloody sputum were classified as pulmonary symptoms, whereas numbness and pain in the body, dysphagia, hoarseness, weakness, and night sweats were classified as non-pulmonary symptoms. The patients were grouped and analyzed according to the duration of their symptoms being shorter or longer than 6 months and their weight loss being more or less than 10%.

The patients’ smoking and drinking habits at the time of the diagnosis were investigated and the pack-years variable was used to evaluate smokers, while the exposure variable was used to evaluate drinkers.

The presence of a second primary cancer in patients was studied in 3 groups according to the presence of the second primary cancer before, after, or concurrent with lung cancer.

The performance status of the patients at the time of the diagnosis was classified according to the ECOG (Eastern Cooperative Oncology Group) scale of performance status [7]. The treatments that the patients received during follow-up were classified as surgery (palliative-radical), chemotherapy (CT; neoadjuvant, adjuvant and palliative), radiotherapy (RT; palliative-curative), and chemoradiotherapy (CRT).

The tumors were classified according to their histological subtypes. Due to the ability of large-cell carcinomas to represent weak or undifferentiated forms of other cancers and the criteria for diagnosing large-cell carcinomas being highly variable, this study focused on squamous cell carcinoma and adenocarcinoma subtypes of NSCLC. Cases of large cell carcinoma and NSCLC – not otherwise specified – were combined and evaluated as ‘other types’. The cancer stage at the time of the diagnosis was recorded. The pathologic stage was taken as a basis in operated patients, while clinical staging was taken as a basis in nonoperated patients. The American Joint Committee on Cancer (AJCC) 8th edition TNM staging system was used for staging the cancer [8]. Stage 1 and stage 2 were combined due to the insufficient number of patients. Stages 1a, 1b, 2a, and 2b were classified as ‘early’, stages 3a, 3b, and 3c were classified as ‘locally advanced’, and stages 4a and 4b were classified as ‘advanced stage’.

Localization, the location of metastasis (if any), the date of pathologic diagnosis, and the diagnosis method of the tumor were recorded from patient files.

The duration of patient follow-up at our clinic, the date of pathological diagnosis, and the date of the last follow-up appointment were determined. Records were made with regard to the last follow-up appointment of the patient if he/she was alive, the time of death if the patient had died, and whether or not the death was related to lung cancer.

Overall survival was defined as the length of time from the date of initial diagnosis to the date of file screening (October 2018) for living patients, and as the length of time from the date of initial diagnosis to the date of death for deceased patients.

### 2.4. Statistical analysis

Statistical analyses were performed using a software package. The effect of predisposing factors on survival was calculated using the log-rank test and survival rates were calculated using the Kaplan–Meier survival analysis. Using the possible factors identified in the previous analyses, independent factors in predicting survival were examined with the backward LR selection method using the Cox regression analysis in the multivariate analysis. P < 0.05 was considered statistically significant.

## 3. Results

The mean age of the patients was 65.39 ± 9.39 years (min. 28 years – max. 87 years). Among the patients, 71.8% (n = 372) were 60 years and older at the time of the diagnosis, and 88.6% (n = 459) of the patients were male. The most common presenting symptoms were pulmonary symptoms (62.7%) (n = 325) and the symptoms were generally present for less than 6 months (80.1%). Moreover, 16.6% (n = 86) of the patients had weight loss. The patients who had 10% or more weight loss at the time of the diagnosis constituted 13.1% (n = 68) of the study population. Of the patients with NSCLC, 84.6% (n = 438) had a history of smoking. Patients who had a history of smoking more than 40 pack-years constituted 62.4% (n = 323), while those who had a history of smoking less than 40 pack-years constituted 22.2% (n = 115) of the study population. Furthermore, 15% of the patients had a history of alcohol use and 47.7% of the study population had a history of chronic disease (Table 1).

**Table 1:** Demographic data of the patients.

	NSCLCn (row%)	Median survival(Months) ± SE	P
Age at the time of diagnosis			
<60	146 (28.2%)	17.97 ± 2.69	<0.001
≥60	372 (71.8%)	9.60 ± 0.79	
Sex			
Female	59 (11.4%)	23.50 ± 5.27	0.004
Male	459 (88.6%)	10.77 ± 0.95	
Symptom			
Pulmonary	325 (62.7%)	15.83 ± 1.35	<0.001
Non-pulmonary	109 (21.1%)	8.40 ± 0.70	(Linear)
Both of them	84 (16.2%)	7.50 ± 0.95	
Symptom time			
<6 months	415 (80.1%)	11.70 ± 1.09	0.557
≥6 months	103 (19.9%)	12.23 ± 2.94	
Weight loss			
Yes			
≥10%	68 (13.1%)	5.77 ± 0.81	<0.001
<10%	18 (3.5%)	9.80 ± 2.86	(Linear)
No	432 (83.4%)	13.80 ± 1.33	
Smoking history			
Yes			
P < 40	115 (22.2%)	11.77 ± 2.13	0.493
P ≥ 40	323 (62.4%)	10.77 ± 0.95	
No	80 (15.4%)	16.20 ± 3.05	
Alcohol history			
Yes	79 (15.3%)	12.37 ± 2.32	0.366
No	439 (84.7%)	11.77 ± 1.10	
Chronic disease			
Yes	247 (47.7%)	12.23 ± 1.88	0.706
No	271 (52.3%)	11.50 ± 1.12	
Overall	518 (100%)	11.77 ± 1.00	

A total of 518 patients who received a histopathological diagnosis were evaluated. Of these patients, 260 (50.1%) were diagnosed with squamous cell carcinoma, 207 (40%) were diagnosed with adenocarcinoma, 50 (9.7%) were diagnosed with NSCLC-NOS, and one patient (0.2%) was diagnosed with large cell carcinoma. According to the performance status of the patients measured when they first presented to the hospital, 80.1% (n = 415) of the patients had a performance status of 2 and below. There were 12 (2.3%) stage 1 patients, 57 (11.0%) stage 2 patients, 171 (33.0%) stage 3 patients, and 278 (53.7%) stage 4 patients. Among the stage 4 patients, 97 patients had multiple organ metastases. When metastatic localizations were analyzed, the most common metastasis was found to be bone metastasis which was present in 158 patients, followed by liver metastasis in 53 patients, adrenal metastasis in 44 patients, contralateral lung metastasis in 43 patients, brain metastasis in 40 patients, pleural-pericardial metastasis in 32 patients, and abdominal metastasis (other abdominal organs except liver and adrenal) in 20 patients, respectively (Table 2). The mean follow-up duration of the patients was 12.63 ± 15.45 months (min: 0–max: 95.83).

**Table 2:** Clinicopathological data of the patients.

	NSCLCn (row%)	Median survival(months) ± SE	P
NSCLC	518	11.77 ± 1.00	
Adenocarcinoma	207 (40%)	12.60 ± 1.59	0.385
Squamous	260 (50.1%)	11.50 ± 1.40	
Other	51 (9.9%)	8.70 ± 1.87	
ECOG			
≤2	415 (80.1%)	15.90 ± 1.41	<0.001
>2	103 (19.9%)	3.53 ± 0.63	
Stage			
1-2	69 (13.3%)	44.47 ± 10.95	
3a	79 (15.2%)	24.60 ± 3.09	<0.001
3b	76 (14.7%)	16.27 ± 2.91	(Linear)
3c	16 (3.1%)	16.13 ± 4.53	
4a	181 (35.0%)	8.07 ± 0.63	
4b	97 (18.7%)	4.33 ± 0.42	
Metastasis			
No	240 (46.3%)	23.63 ± 1.50	<0.001
Single	181 (35.0%)	8.07 ± 0.63	(Linear)
≥2	97 (18.7%)	4.33 ± 0.42	
Metastasis Location*			
Bone met.			
Yes	158 (56.8%)	4.90 ± 0.75	0.003
No	120 (43.2%)	8.07 ± 0.70	
Liver			
Yes	53 (19.1%)	4.43 ± 0.50	<0.001
No	225 (80.9%)	7.70 ± 0.68	
Brain			
Yes	40 (14.4%)	4.33 ± 1.61	0.762
No	238 (85.6%)	7.07 ± 0.55	
Adrenal			
Yes	44 (15.8%)	5.57 ± 1.33	0.148
No	234 (84.2%)	7.03 ± 0.59	
Contralateral Lung			
Yes	43 (15.5%)	8.40 ± 1.44	0.552
No	235 (84.5%)	6.33 ± 0.62	
Pleural/pericardial			
Yes	32 (11.5%)	9.03 ± 1.41	0.018
No	246 (88.5)	6.20 ± 0.55	
Abdomen			
Yes	20 (7.2%)	5.37 ± 1.57	0.813
No	258 (92.8%)	7.03 ± 0.58	
Tumor localization			
Right	296 (57.2%)	11.77 ± 1.34	0.397
Left	222 (42.8%)	11.47 ± 1.47	

*Only metastasis group.

The patients were evaluated with the treatments they received during follow-up. Since 123 patients were not followed-up at our clinic (93 patients applied to another medical center and 30 patients refused treatment), these patients were analyzed as the group with unknown treatment status when evaluating treatments. Considering the surgical treatments, 62 patients underwent radical and 10 patients underwent palliative surgery, while 323 patients did not receive surgical treatment and 123 patients were not followed-up at our clinic. Furthermore, 215 patients had received systemic CT. Among these patients, 12 received neoadjuvant, 41 received adjuvant, and 166 received palliative therapy. Since 4 patients received neoadjuvant therapy followed by adjuvant therapy, the group that received neoadjuvant and adjuvant therapy consisted of 49 patients. Of the 42 patients who received RT, 16 patients were given adjuvant RT and 26 patients were given curative RT. There were 95 patients who received CRT treatment (Table 3).

**Table 3:** Treatment information of the patients.

	NSCLCn (row%)	Median survival(months) ± SE	P
Surgery			
Radical Surgery	62 (12.0%)	68.94 ± 5.92*	<0.001**
Palliative Surgery	10 (1.9%)	7.53 ± 2.00	
No Surgery	323 (62.4%)	12.03 ± 0.89	
Unknown	123 (23.7%)	5.10 ± 0.74	
Chemotherapy			
Neoadjuvant/Adjuvant CT	49 (9.5%)	64.79 ± 6.98*	<0.001***
Palliative CT	166 (32.1%)	11.10 ± 0.94	
Those who did not receive chemotherapy	180 (34.7%)	13.43 ± 1.63	
Unknown group	123 (23.7%)	5.10 ± 0.74	
Radiotherapy			
Adjuvant RT	16 (3.1%)	45.50 ± 12.68	<0.001****
Curative RT	26 (5.1%)	14.37 ± 3.05	
Those who did not receive RT	353 (68.1%)	13.43 ± 1.27	
Unknown group	123 (23.7%)	5.10 ± 0.74	
CRT			
Those who received CRT	95 (18.4%)	18.63 ± 1.84	<0.001*****
Those who did not receive CRT	300 (57.9%)	12.20 ± 1.11	
Unknown group	123 (23.7%)	5.10 ± 0.74	

*Mean (when median cannot be calculated). **Difference: 1. A difference (P < 0.001, P < 0.001, P < 0.001, respectively) was found between the group that received radical surgical treatment and the groups that received palliative surgical treatment, no surgical treatment and the unknown group. 2. A difference (P < 0.001) was found between the unknown group and the group that did not receive surgical treatment. ***Difference: 1. A difference (P < 0.001, P < 0.001, P < 0.001, respectively) was found between the group that received neoadjuvant/adjuvant CT treatment and the group that received palliative CT, the group that did not receive CT and the unknown group. 2. A difference (P < 0.001, P < 0.001, respectively) was found between the unknown group and the group that received palliative CT and the group that did not receive CT. ****Difference: 1. A difference (P < 0.001, P = 0.001, P < 0.001, respectively) was found between the group that received adjuvant RT and the groups that received curative RT, did not receive RT and the unknown group. 2. A difference (P < 0.001) was found between the group that did not receive RT treatment and the unknown group. *****Difference: 1. A difference (P = 0.046) was found between the group that received CRT and the group that did not receive CRT. 2. A difference (P < 0.001, P < 0.001, respectively) was found between the unknown group and the group that received CRT and the group that did not receive CRT.

### 3.1. Univariate survival analysis

The results of univariate analyses revealed that the patient’s age being below 60 years (P < 0.001) and sex being female (P = 0.004) significantly increased the survival time. When the presence of symptoms at the time of the diagnosis were ranked as pulmonary, nonpulmonary, and both, and weight loss was ranked as none, less than 10%, and more than 10%, the survival time was found to decrease in proportion to the rank (linear P < 0.001, P < 0.001, respectively) (Table 1).

The median survival was found to be 11.77 ± 1.00 months in all patients diagnosed with NSCLC, with a median life expectancy of 11.50 ± 1.40 months in patients with squamous cell carcinoma, 12.60 ± 1.59 months in patients with adenocarcinoma, and 8.70 ± 1.87 months in other patients. The 5-year relative survival rate for NSCLC was 8% (7% for men and 18% for women). No significant difference was detected in terms of the survival time between histopathological subtypes (Table 2).

The patient having an ECOG performance status above 2 at the time of the diagnosis significantly reduced the survival time (P < 0.001). It was found that the survival time significantly decreased with each stage increase (linear P < 0.001). The analysis of the patients with no metastasis, single metastasis, and multiple metastases revealed that the survival times were significantly reduced in the same order (linear P < 0.001) and that bone, liver, and pleural-pericardial metastases had a significant effect on survival times (P = 0.003, P < 0.001, P = 0.018, respectively) (Table 2).

When the patients were evaluated according to the treatments they received, it was found that the survival times of the patients who received radical surgical treatment was significantly longer than the patients who received palliative surgical treatment and patients who did not receive surgical treatment (P < 0.001, P < 0.001, respectively). In terms of the CT treatment, it was observed that the patients who received neoadjuvant/adjuvant CT had significantly longer survival times than the patients who received palliative CT and patients who did not receive CT (P < 0.001, P < 0.001, respectively) (Table 3). When the study population was evaluated in terms of RT treatment, it was found that those who received adjuvant RT treatment had longer survival times compared to the patients who received palliative RT treatment and patients who did not receive RT treatment (P < 0.001, P = 0.001, respectively). The survival times of the patients who received CRT treatment were significantly longer than the patients who did not receive CRT treatment (P = 0.046) (Table 3).

In order to better evaluate the effects of the treatments received by the patients, the effect of the treatment on survival was calculated using a separate log-rank analysis by making a correction according to the stage of cancer. The significantly high survival times in patients that underwent radical surgery was also observed in the early stage cancer and locally advanced cancer groups (Table 4).

**Table 4:** Kaplan–Meier Median Survival of the patients with non-small cell lung cancer (NSCLC) according to stage and treatment.

	Early cancers(stage 1-2)	Locally advanced(stage 3)	Metastatic cancer(stage 4)	P
Surgery				
Radical surgery	71.96 ± 7.27*	55.97 ± 8.78	-	<0.001**
Palliative surgery	-	18.43 ± 0.01	7.53 ± 2.93	
No surgery	20.23 ± 3.99	17.70 ± 1.19	8.57 ± 0.62	
Unknown group	24.57 ± 8.25	16.27 ± 3.34	3.60 ± 0.51	
Chemotherapy				
Neoadjuvant/Adjuvant CT	41.87 ± 12.06	71.42 ± 9.43*	-	<0.001***
Palliative CT	-	18.43 ± 8.41	10.77 ± 1.42	
Those who did not receive CT treatment	58.50 ± 11.08	17.70 ± 1.25	7.99 ± 3.60	
Unknown group	24.57 ± 8.25	16.27 ± 3.34	3.60 ± 0.51	
Radiotherapy				
Adjuvant RT	28.23 ± 2.24	45.50 ± 11.25	-	<0.001****
Curative RT	17.47 ± 3.54	14.36 ± 2.96	-	
Those who did not receive RT treatment	63.57 ± 14.04	21.27 ± 2.74	8.10 ± 0.56	
Unknown group	24.57 ± 8.25	16.27 ± 3.34	3.60 ± 0.51	
CRT				
Those who received CRT treatment	44.47 ± 17.08	18.63 ± 1.84	-	<0.001*****
Those who did not receive CRT treatment	58.50 ± 6.55	24.03 ± 6.02	8.10 ± 0.56	
Unknown group	24.57 ± 8.25	16.27 ± 3.34	3.60 ± 0.51	

*Mean (when median cannot be calculated). **Difference: 1. A difference (P <0.001) was found between the group that did not receive surgical treatment and the unknown group. 2. A difference (P = 0.001, P = 0.029, respectively) was found between the group that received radical surgical treatment and the group that did not receive surgical treatment and the unknown group. ***Difference: 1. For the locally advanced group, a difference (P = 0.003, P < 0.001, P < 0.001, respectively) was found between the group that received Neoadjuvant/Adjuvant CT treatment and the group that received palliative CT treatment, the group that did not receive CT treatment and the unknown group. 2. For the metastatic cancer group, a difference (P < 0.001, P < 0.001, respectively) was found between the group that received palliative CT treatment and the group that did not receive CT treatment. ****Difference: 1. A difference (P < 0.001) was found between the group that did not receive RT treatment and the unknown group. 2. A difference (P = 0.011, P = 0.012, respectively) was found between the group that received curative RT treatment and the group that received adjuvant RT treatment and the group that did not receive RT treatment. 3. For the locally advanced group, a difference (P < 0.001, P = 0.018, respectively) was found between the group that received Adjuvant RT treatment and the group that did not receive RT treatment and the unknown group. *****Difference: A difference (P < 0.001, P < 0.001, respectively) was found between the unknown group and the group that did not receive CRT treatment and the group that received CRT treatment.

Considering the chemotherapy treatment, it was observed that in the locally advanced group, the patients who received neoadjuvant/adjuvant CT treatment had significantly longer survival times than the patients who received palliative CT and patients who did not receive CT treatment (P = 0.003, P < 0.001, respectively). In the metastatic cancer group, the patients who received palliative CT treatment had significantly longer survival times than the patients who did not receive CT treatment (P < 0.001).

Evaluation of the RT treatment according to the stages in all groups showed that the patients who received adjuvant RT treatment had a significantly longer survival time than the patients who received curative RT treatment, while the patients who did not receive RT treatment had a significantly longer survival time than the patients who received curative RT treatment (P = 0.011, P = 0.012, respectively). In addition, it was observed in the locally advanced group that the patients who received adjuvant RT treatment had a longer survival time than the patients who did not receive RT treatment (P < 0.001) (Table 4).

The evaluation of the CRT treatment according to the stages showed that there was no difference between the patients who received CRT treatment and patients who did not receive CRT treatment (Table 4).

### 3.2. Multivariate survival analysis

The Cox proportional hazards model was used in this study to eliminate the complexity of and the interactions between predictive values affecting survival and survival times in lung cancer and to make the results more reliable. The multivariate analysis included the parameters of age, sex, the presence of symptoms, weight loss, ECOG performance, stage, the presence of adrenal, bone, pleural-pericardial, or liver metastases, whether or not the patient received chemotherapy, radiotherapy, surgical, or chemoradiotherapy treatment, which were found to be significant in univariate analyses, which had a P value that was close to a type 1 error value (with a cut-off value of P = 0.25) and which had a correlation coefficient below 0.6 according to the matrix of regression coefficients.

**Figure 2:**
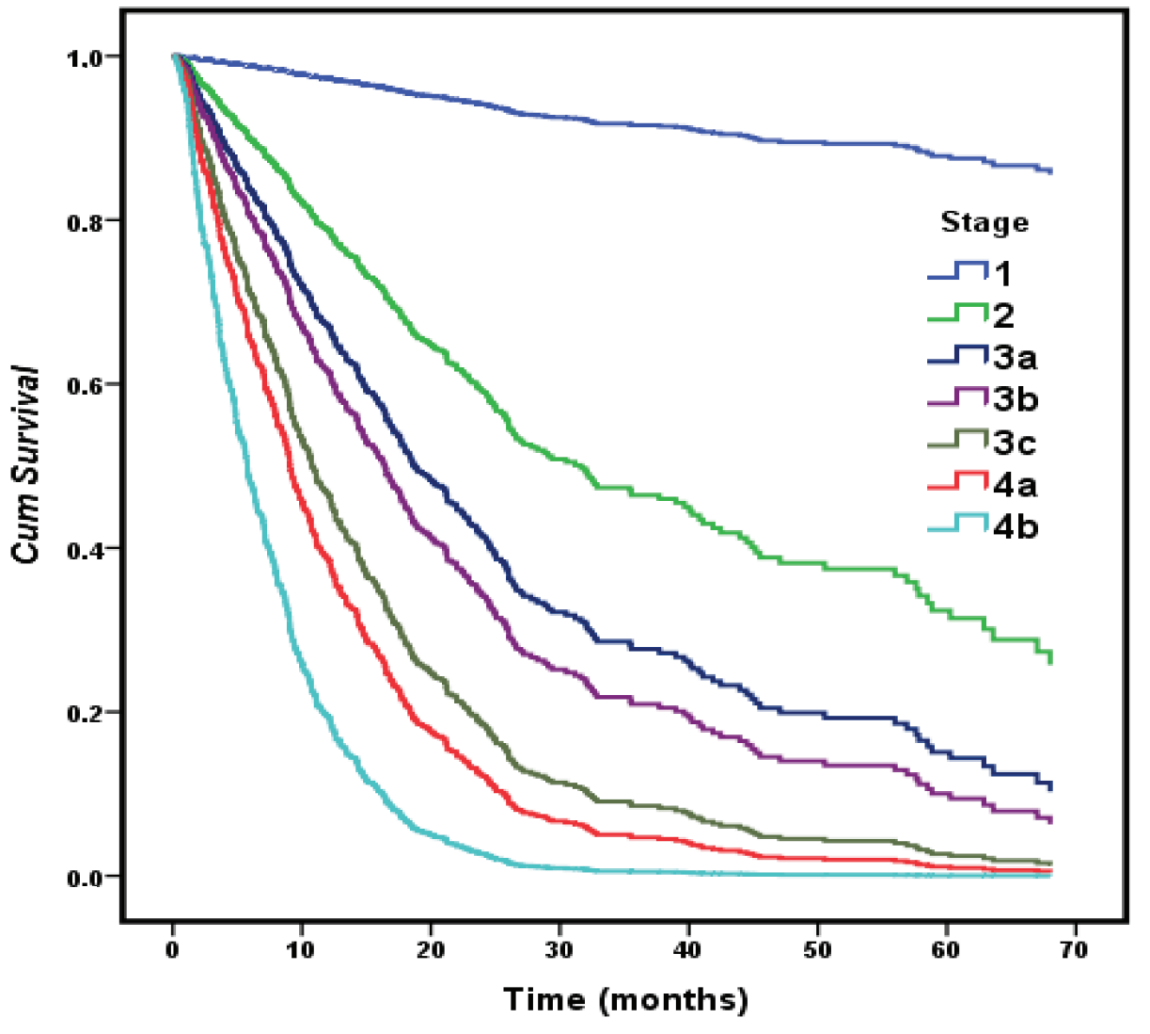
Cox regression survival curves with the effect of cancer stage.

**Figure 3:**
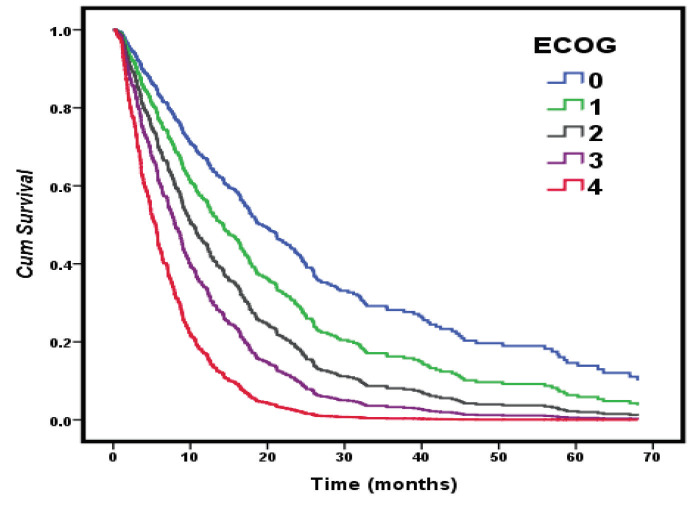
Cox regression survival curves with the effect of ECOG.

In the multivariate analysis, male sex (HR, 2.4; P < 0.001), ECOG > 2 (HR, 1.70; P < 0.001), stage (HR, 1.37; P = 0.045), the presence of bone or liver metastasis (HR, 1.44; P = 0.009, HR, 1.57; P = 0.016), and the patient not having received radiotherapy (HR, 3.25; P < 0.001) or chemotherapy (HR, 1.85; P = 0.001) treatment were defined as statistically significant predictive factors that reduced the overall survival. One unit of increase in the stage of cancer (1-2/3a/3b/3c/4a/4b) increased the risk of survival decrement by 1.367-fold (Table 5, Figure 2, Figure 3).

**Table 5:** Survival analysis for OS in non-small cell lung cancer.

Covariates	Hazard Ratio	95% CI	P value
Sex			
Male versus female	2.409	1.609–3.605	<0.001
ECOG			
> 2 versus ≤ 2	1.702	1.264–2.292	<0.001
Stage*(per 1 number increase)	1.367	1.006–1.857	0.045
Bone met.			
Yes versus No	1.444	1.097–1.900	0.009
Liver			
Yes versus No	1.569	1.088–2.262	0.016
RT*(per 1 number increase)			
Adjuvant RT/Curative RT/ RT not received/Unknown	3.251	2.378–4.445	<0.001
CT*(per 1 number increase)			
Neoadjuvant-Adjuvant CT/CT not received/Palliative CT/Unknown group	1.847	1.307–2.610	0.001

* linear OS: overall survival Method: Backward Stepwise (likelihood ratio): -2 Log likelihood: 2451.264; Omnibus test of model coefficients: P = 0.000.

## 4. Discussion

According to the GLOBOCAN 2018 data, the 5-year survival rate for lung cancer is 18% and only 16% of lung cancer cases are diagnosed at an early stage. In this study, the 5-year relative survival rate for NSCLC was 8% (7% for men and 18% for women) and 13.3% of the patients were diagnosed at an early stage (stage 1 and stage 2). The 5-year survival rate of patients diagnosed at an early stage (stage1-2) was 34%, whereas the 5-year survival rate of patients diagnosed at a locally advanced and advanced stage (stage 3-4) was 5%. The low survival rates in this study are due to the fact that 86.7% of our patients were diagnosed at a locally advanced and advanced stage. Survival rates in studies conducted with locally advanced and advanced stage patients are similar to the survival rates found in our study [1]. Wang et al. calculated the 5-year survival rate as 6.6% in their study conducted with stage 3 and stage 4 NSCLC patients [9].

In this study, predictive values affecting survival in patients with NSCLC were age, sex, the presence of symptoms, weight loss, ECOG performance, stage, metastasis status, the presence of bone, pleural-pericardial, and liver metastases, and chemotherapy, radiotherapy, surgical, or chemoradiotherapy treatment status according to the univariate analyses. Male sex, an ECOG value >2, increased stage, the presence of bone or liver metastasis, and the patient not having received radiotherapy or chemo radiotherapy treatment were defined as factors that decrease overall survival according to the regression results.

The results were significant in terms of the prediction of outcomes and the effectiveness of the study population. With one unit of increase in the stage of cancer (1-2/3a/3b/3c/4a/4b), the risk increased significantly by 1.367-fold. In many studies conducted on both NSCLC and SCLC, the stage of the disease is accepted as the most important prognostic indicator for the determination of survival [10–12].

Due to the fact that lung cancer survival rate varies according to histological type and due to the differential therapeutic efficacy and toxicity of newly adapted therapies for NSCLC subtypes, a more precise histological subtyping became necessary in order to use the terms adenocarcinoma, squamous cell carcinoma, and NSCLC-NOS [13,14]. Furthermore, the distribution of the histological types and subtypes of microscopically confirmed lung cancer varies between countries. In general, the incidence of adenocarcinoma among men is higher than that of squamous cell carcinoma. Histology-specific survival is higher for adenocarcinoma and squamous cell carcinoma than large cell carcinoma or small cell lung cancer [15]. In our study, the survival time for adenocarcinoma was found to be longer than that for other types of cancer, which was similar to the literature [16]. However, no difference was found between the histological subtypes and this may be due to the subtypes that could not be classified [17,18].

It was found that male had a 2.4-fold higher risk in terms of overall survival and this finding is supported by studies and meta-analyses conducted with patients with NSCLC [11,19,20]. Radzikowska et al. have found in their study conducted with 20,561 cases that low ECOG performance (0-1 versus 3-4) was 2.58 times riskier in terms of reduced survival [11]. In another study examining stage 4 NSCLC patients, the risk was 1.9-fold higher [21]. In this study, low performance status affected the survival significantly and constituted a 1.7-fold risk for overall survival. In addition, the effect of ECOG and Cox regression survival curves provided in Figure 3 shows that a worsening performance scale is associated with poor survival. Performance status and weight loss have been shown to be important prognostic factors for NSCLC [12,22,23]. According to the univariate analysis results in this study, weight loss at the time of the diagnosis significantly reduced the survival time. Although smoking status is a significant prognostic factor for survival according to the literature, its effect could not be shown in this study [24].

In our study, patients with single organ metastasis had significantly longer survival times than patients with multiple organ metastases, which is also supported in the literature [25]. However, there are inconsistencies in the literature about the sites of metastasis, with some studies reporting that liver or adrenal metastasis is worse, and others reporting that bone metastasis is worse [25,26]. For this reason, among the metastasis status (none-single-multiple) and metastasis sites that showed a high correlation, we deemed it appropriate to include the metastasis sites in the multivariate analyses conducted in our study. According to the regression analysis, bone and liver metastases were 1.4 and 1.6 times riskier in terms of survival, respectively. Bone metastasis is believed to be associated with survival in addition to the pathological processes related to the skeletal system such as pathological fractures and hypercalcemia [27]. Metastatic liver lesions are rarely associated with severe symptoms, but it is known that most NSCLC patients with liver metastasis do not respond well to chemotherapy [28,29]. Finkelstein et al. described bone and liver metastases as independent prognostic factors in 893 metastatic NSCLC patients [30], while Tomohiro et al. supported the negative effects of liver and adrenal gland metastasis on survival with multivariate analysis results [26]. This study also showed that bone and liver metastases were prognostic factors which significantly short survival time in both univariate and multivariate analysis results.

Surgical resection, if possible, remains to be the most consistent and successful option for treatment in patients with NSCLC and provides a chance for long-term survival [31]. The survival time of the patients, who were diagnosed at an early and locally advanced stage and were able to undergo radical surgery, was significantly longer in this study. This result may be remarkable in terms of showing the effect of radical surgical treatment in patients with locally advanced lung cancer.

Most of the phase 3 studies have shown that systemic chemotherapy is superior to the best supportive care in locally advanced and metastatic lung cancer patients [32]. Some meta-analyses have also supported that CT treatment in advanced NSCLC patients provides improved survival compared to supportive care [33–35]. It was found in this study that advanced stage patients who received palliative CT treatment had a significantly longer survival time than those who did not receive CT treatment. In addition, the efficacy of CT treatment was also significant according to the results of further analyses.

The first major research on the role of RT in the treatment of lung cancer was conducted by the Veterans Administration Lung Study Group, wherein RT and placebo groups were compared among patients with small cell and non-small cell lung cancer, and higher survival rates were found in patients who received RT treatment [36,37]. In both the univariate and advanced analyses performed in this study, the patients who did not receive RT treatment were found to have a 2.45-fold higher risk in terms of overall survival compared to the patients who received adjuvant RT and curative RT.

Studies have shown that CRT treatment improves survival in patients with non-small cell lung cancer [38,39]. In this study, the efficacy of CRT was observed in the univariate analyses that were performed, but the same effect could not be demonstrated in advanced analyses.

The survival times of the patients who did not receive CT treatment, RT treatment, and CRT treatment at an early stage were longer than those of the patients who received these treatments. This is due to the fact that the patient group that did not receive CT treatment also encompassed the group that received other treatments (e.g., RT, surgery, CRT). Additionally, this study does not include comprehensive information such as treatment protocols and laboratory tests since it was not designed to evaluate the effect of treatment on patients with NSCLC.

In conclusion, a median survival of 11.77 ± 1.00 months and a 5-year relative survival rate of 8% were found in this study for patients diagnosed with NSCLC. Univariate analyses revealed that the patient’s age, sex, weight loss, the presence of pulmonary and non-pulmonary symptoms, performance scale, stage, metastasis status (none-single-multiple), bone, liver, and pleural-pericardial metastasis and treatments (surgery, CT, RT, CRT) were prognostic factors that significantly affected the survival time. Sex, stage, performance status, the presence of liver or bone metastasis, and RT and CT treatments were shown to have an effect on overall survival in multivariate analysis. Further intervention studies are needed for the variables determined as a result of multivariate analysis.
